# Disturbance and Plant Succession in the Mojave and Sonoran Deserts of the American Southwest

**DOI:** 10.3390/ijerph7041248

**Published:** 2010-03-25

**Authors:** Scott R. Abella

**Affiliations:** School of Environmental and Public Affairs, University of Nevada Las Vegas, Las Vegas, NV 89154-4030, USA; E-Mail: scott.abella@unlv.edu; Tel.: +1-702-895-5163; Fax: +1-702-895-4436

**Keywords:** arid land, recovery, revegetation, fire, management, resource damage, dust mitigation, diversity

## Abstract

Disturbances such as fire, land clearing, and road building remove vegetation and can have major influences on public health through effects on air quality, aesthetics, recreational opportunities, natural resource availability, and economics. Plant recovery and succession following disturbance are poorly understood in arid lands relative to more temperate regions. This study quantitatively reviewed vegetation reestablishment following a variety of disturbances in the Mojave and Sonoran Deserts of southwestern North America. A total of 47 studies met inclusion criteria for the review. The time estimated by 29 individual studies for full reestablishment of total perennial plant cover was 76 years. Although long, this time was shorter than an estimated 215 years (among 31 individual studies) required for the recovery of species composition typical of undisturbed areas, assuming that recovery remains linear following the longest time since disturbance measurement made by the studies.

## 1. Introduction

**Figure 1.**
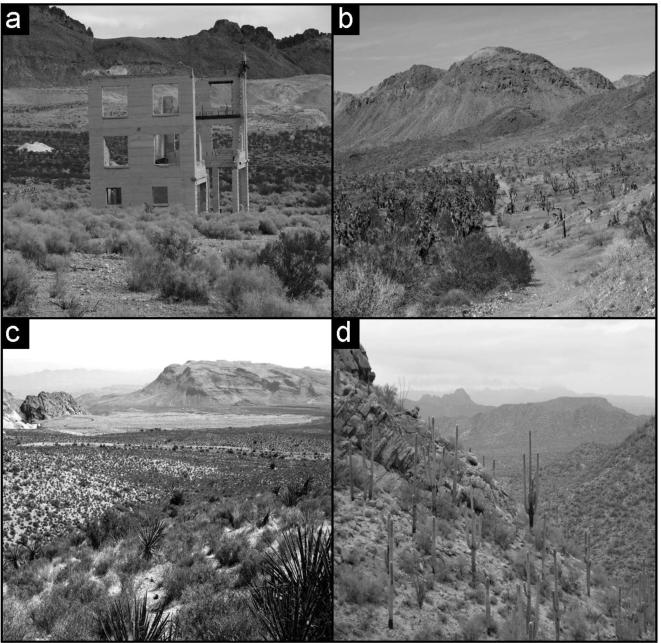
**(a)** View of part of the ghost town of Rhyolite, Nevada, in the Mojave Desert, showing distinct bands of recovering disturbed plant communities (foreground), relatively undisturbed creosote bush communities (center band of darker vegetation), and a de-vegetated band disturbed by mining in the middle-top of the photograph. **(b)** The 2005 Tramp Fire in the eastern Mojave Desert showing clearly demarcated burned habitat (right) and unburned Joshua tree woodland (left). **(c)** With its light color in the middle-top of the photo, the 2005 Loop Fire west of Las Vegas, Nevada, in the Mojave Desert illustrates the landscape-scale scars created by disturbance in the desert. **(d)** Example of undisturbed desert habitat containing the columnar cactus giant saguaro in the Sonoran Desert uplands of Saguaro National Park, Arizona. Photos by S.R. Abella in 2006 (a), 2007 (c), and 2008 (d), and by E.C. Engel in 2008 (b).

Humans have been extensively disturbing the environment of the hot deserts of the American Southwest since the mid-1800s (Figure 1). Some of these disturbances have facilitated, or attempted to facilitate, widespread public benefits, but not without environmental costs. Mining, for example, has exported natural resources from the region since the 1800s and remains prevalent today, producing materials such as gypsum, cinders, gold, and copper used for a variety of societal products [1]. Water and energy transmission corridors carry resources within and through the region, yet result in long, linear areas of cleared disturbed land [2]. Dry-land agriculture provided brief booms to local settlements in the early and mid-1900s, and while agriculture continues on a limited basis today, abandoned fields have left a legacy of de-vegetated lands [3,4]. Road building has enabled access to large tracts of public land for recreational use. However, proliferation of roads and unauthorized off-road vehicle use has left persistent scars in the desert [5]. For example, in their analysis of the road network in the 6,475-km^2^ Mojave National Preserve in southern California, Vogel and Hughson [6] found that roads proliferated from 605 km in total length in 1885 to 3,701 km in 1994. The US military extensively used the deserts for World War II training operations, and its largest training facilities still reside in southwestern deserts [7,8]. Intensive clearing of the desert also has occurred for human settlements, some of which were abandoned in the early 1900s (often following brief mining booms) to become ghost towns with dirt street systems still clearly visible [9]. Today, two of the largest cities in the USA (Las Vegas, Nevada, metropolitan area with 1.9 million people, and Phoenix, Arizona, with 4.3 million people) are located in southwestern deserts.

There are environmental and public health costs associated with some of the benefits of these disturbances, however. The disturbances remove plant cover, which can negatively impact wildlife species, such as desert tortoise (*Gopherus agassizii*) listed under the USA Endangered Species Act [10]. De-vegetated areas can incur severe soil wind erosion, releasing fugitive dust as air pollution that can be a serious public health hazard. Grantz *et al*. [11] found that air quality standards for particulate matter, which causes respiratory problems in humans, were breached for 11−25 days/year downwind of abandoned agricultural land in the western Mojave Desert of California. Blowing sand also disrupted airport operations and resulted in hazardous driving conditions. These immediate effects on public health are in addition to longer term effects associated with soil loss, including reduced potential for agriculture, carbon sequestration, and other land uses.

Fires, not considered common historically in southwestern deserts but increasing in extent in recent decades partly because of fuel provided by non-native annual grasses (e.g., red brome [*Bromus rubens*] and buffelgrass [*Pennisetum ciliare*]), are having significant economic and environmental impacts [12]. Fire has killed the charismatic Joshua tree (*Yucca brevifolia*) in areas of Joshua Tree National Park in California and the renowned giant saguaro cactus (*Carnegiea gigantea*) in Saguaro National Park in Arizona [13,14]. Joshua tree and saguaro are not considered well adapted to fire (which was not part of their evolutionary environment), as fire readily kills them and they rarely resprout [15]. These species often require protection (e.g., shading) by existing vegetation (“nurse plants”) from the harsh desert environment for reproduction, so regeneration of new individuals is slow because the nurse plants must first become established after fire [13]. Areas containing these species are national and international tourist destinations. From an economic standpoint, the tourism industry (e.g., resorts, golf courses, businesses dependent on visitation to area parks) is concerned about having scenery and tourist attractions disrupted [14]. Furthermore, fires have threatened human habitations and cost millions of dollars to suppress, such as the 100,000 ha Cave Creek Fire in 2005 near Phoenix, Arizona, in the Sonoran Desert [16].

Most disturbed areas in the desert are simply left to natural recovery processes, rather than being revegetated through active management treatments such as seeding, planting, or soil manipulation [17]. Active revegetation has shown some success in southwestern deserts for enhancing and accelerating recovery [3]. However, revegetation has generally been confined to small areas because of its expense, logistical challenges associated with implementing treatments across vast desert regions, and unpredictable weather that makes effectiveness uncertain [18]. Understanding natural vegetation reestablishment after disturbance is important for several reasons. First, determining whether natural reestablishment will meet environmental management objectives (e.g., for minimizing dust pollution, promoting wildlife habitat or aesthetics) can assist decision-making on whether attempting active revegetation is desired or worth the expense. Second, understanding natural recovery could help inform how to make active revegetation more effective by mimicking natural processes. Third, knowledge of recovery can allow estimates of how long original plant communities may take to reestablish, such as Joshua tree and saguaro communities, or even if they will fully recover since present climates may differ from the past evolutionary environments of the species. This type of information can inform management decisions such as where to attempt the most aggressive fire suppression to protect resources from disturbance in the first place to avoid long recovery times.

There is a body of theory on succession in ecological and environmental science that may help provide a framework for understanding changes induced by disturbance in desert ecosystems. The process of biological communities becoming established following disturbance is termed succession by ecologists and is differentiated into primary and secondary succession [19]. Primary succession occurs on newly created geomorphic surfaces (e.g., volcanic islands surfacing in oceans, or debris flows forming alluvial fans in deserts) not previously containing vegetation. Secondary succession occurs in areas that were vegetated prior to a disturbance. Disturbance is defined as a physical force (e.g., hurricane, fire, road building) that removes most or all of plant biomass. Ecologists have had various interpretations of the definitions of succession, ranging simply from changes in biological communities over time, to directional changes where communities pass through relatively distinct post-disturbance seral stages that culminate in the reestablishment of the pre-disturbance community [5,20]. In this paper, post-disturbance recovery is defined as the return of any variable (e.g., plant cover, or the number of species termed species richness within a defined area) to those of nearby levels found on undisturbed areas. Succession is considered to have occurred if there is at least one intermediary community that becomes established after disturbance followed by reestablishment of communities resembling adjacent undisturbed areas.

Succession in arid lands has long puzzled ecologists and environmental managers. For example, in 1940, Muller [21] concluded that no succession occurred in creosote bush (*Larrea tridentata*) Chihuahuan Desert shrub communities of western Texas following soil disturbance because the early colonizers had been components of the previous late-successional community occupying the site. In 1942, Shreve [22] also concluded that pre-disturbance species were the initial colonizers of disturbed areas in the Sonoran Desert, and that true succession was absent because the early colonizers did not alter the environment to facilitate colonization by later species. In contrast, in 1961 Wells [23] examined a 33-year-old ghost town in the Mojave Desert of Nevada and observed that pioneer species (different from the surrounding undisturbed vegetation) had colonized the site and succession had occurred, though insufficient time had elapsed to determine if the full undisturbed community would become established. Similarly, in 1979, Vasek [24] surmised that succession occurred on a cleared roadside borrow pit area in California’s Mojave Desert, a claim disputed by Rowlands in 1980 [5], who believed that data from a single site (which may have differed in soil properties, confounding vegetation patterns) did not demonstrate succession. Webb *et al*. [20] noted that some of the confusion about arid land succession resulted from unclear definitions of succession and recovery, limited field data, uncertainty in applying to deserts successional concepts from moister regions where succession is better understood, and the slow rates of change in perennial plant communities in deserts. This slower scale makes it difficult for researchers to study changes in deserts on the typical short research funding cycles and poses logistical challenges for finding sites that have had sufficient time to actually record a succession [25,26]. More recently, a variety of studies in American Southwest deserts have examined plant establishment after disturbances (e.g., [27–29]), but this literature is fragmented and may benefit from a synthesis evaluating evidence for the occurrence of general concepts and patterns.

Several concepts have been advanced in the literature about succession in deserts. Disturbance type has been theorized to influence succession, where the most severe disturbances (e.g., those that remove surface soil layers such as clearing by bulldozers), or those that heavily compact soils, retard succession and recovery [30]. Plant community type (e.g., creosote bush versus blackbrush [*Coleogyne ramosissima*] communities) also is suggested to affect succession, with some community types recovering more quickly than others after disturbance [31]. Webb *et al*. [20] noted that the age of the previous community (such as those established by primary succession then being disturbed to invoke secondary succession) can influence succession, generally with the older the community, the longer the recovery time. Vasek [32] concluded that unlike in temperate regions where annual plants often comprise the initial seres after disturbance, annuals occur throughout succession in deserts because annuals are prominent components of mature communities. Several authors noted that early successional perennial plants usually have short life spans and often inhabit washes, which are disturbed naturally by periodic floods [23,24,33]. Some have hypothesized that if disturbed, the oldest communities may not actually recover and succession will proceed to alternative stable states [20]. The reasoning is that climate and other conditions (e.g., invasion of exotic species, anthropogenic N deposition) have changed since the communities developed, so another stable community will become established instead of the original community. Synthesizing data from a variety of studies may provide insight into how general these suppositions may be in a variety of environmental settings.

The purpose of this study was to synthesize the status of knowledge of disturbance effects on vegetation and post-disturbance recovery and succession in the Mojave and Sonoran Deserts of the American Southwest. A systematic, quantitative review approach was employed. Specific questions examined included:

1. What is the relationship of plant community cover, species richness, and species composition with time since disturbance (TSD), and do these measures change at different rates?
2. Are there differences in successional pattern between primary and secondary succession and among disturbance and community types in secondary succession?
3. Which species are dominant early successional colonizers?
4. Do successional patterns differ between annual and perennial species?
5. Is there evidence supporting generalizations proposed in the literature about arid land succession, such as successional sequences being similar to more temperate regions but requiring longer time periods to develop?

## 2. Methods

### 2.1. Study Area

**Figure 2.**
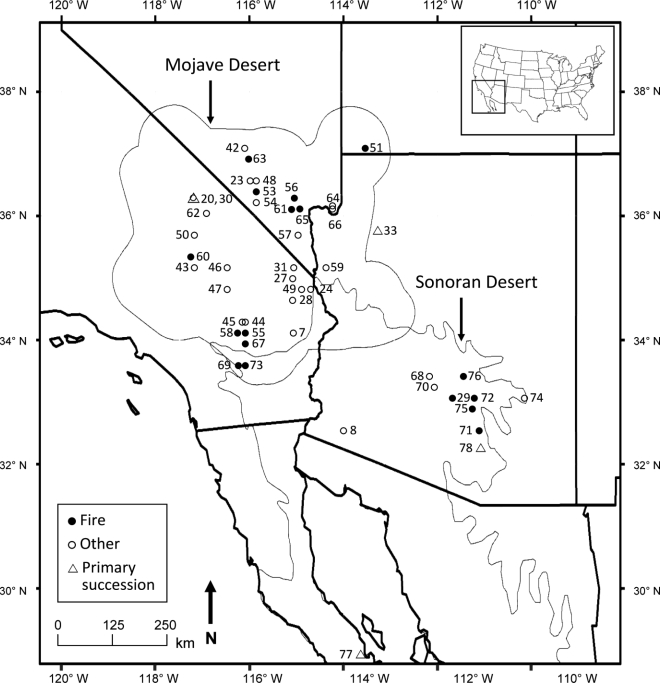
Location of 47 studies meeting selection criteria for a quantitative analysis of plant recovery and succession in the Mojave and Sonoran Deserts of the American Southwest. Studies are distinguished by disturbance type (fire or land-clearing disturbances such as roads) and are numbered according to the Appendix and References Section.

The Mojave Desert, approximately 124,000 km^2^ in size [34], occupies parts of California, Nevada, Utah, and Arizona in the southwestern USA (Figure 2). About 310,000 km^2^ in size, the Sonoran Desert is in parts of California and Arizona, USA, and Sonora and Baja California, Mexico [35]. The two deserts share a boundary at the southeastern part of the Mojave and the northwestern part of the Sonoran. Both are classified as warm deserts, although the Mojave has cooler winter temperatures while the Sonoran has more of a subtropical climate [36]. Much precipitation occurs in winter in the Mojave Desert, whereas precipitation is more bimodal (summer and winter) or evenly distributed throughout the year in the Sonoran Desert. An example weather station (Las Vegas, Nevada, USA, 662 m elevation) in the eastern Mojave Desert has reported averages of 11 cm/yr of precipitation, July daily maximum temperature of 40 °C, and January daily minimum temperature of 1 °C (1937–2008 records; [37]). At Ajo, Arizona, at a comparable weather station (elevation 549 m) in the north-central Sonoran Desert, averages of 21 cm/yr of precipitation, 39 °C July daily maximum, and 5°C January daily minimum have been recorded from 1914−2008. Precipitation can vary substantially among years in these deserts, resulting in some years having large blooms of annual plants and other years with few annuals [38].

Topography of the deserts is diverse, including mountain ranges, dry lakes (playas), relatively flat plains, bajadas (alluvial fans or debris flows), washes that are intermittent stream drainages, and various volcanic landforms [39]. Major soil orders, following the US classification system, include Aridisols and Entisols [40]. These different environmental complexes support different plant communities. Some characteristic species that differ between the deserts include the Joshua tree inhabiting the Mojave Desert and the columnar cactus giant saguaro of the Sonoran Desert uplands [39]. Creosote bush shrublands occupy vast areas of plains in both deserts at the lower elevations. The typical vegetation appearance of the deserts is scattered perennial shrubs, cacti, forbs, and grasses, separated by interspaces that consist of sparsely vegetated soil or annual plants during moist years [36]. Perennial shrubs form “fertile islands,” which are microsites containing fertile soils and ameliorated microclimates [41]. These fertile islands often contain the greatest overall concentrations of annual plants, although some annual species are most abundant in interspaces. Livestock grazing (primarily cattle and sheep) was common in the region in the late 1800s to the past several decades [1]. Localized livestock grazing still occurs, as does plant consumption by wild burros (*Equus asinus*) and horses, (neither considered native) and native herbivores such as bighorn sheep (*Ovis canadensis*) and black-tailed jackrabbit (*Lepus californicus*).

### 2.2. Literature Search

The article databases of Academic Search Premier (coverage 1975–present), Agricola (1400s–present), Biological Abstracts (1969–present), JSTOR (individual journals since their inception up to 2005–2006), ScienceDirect (variable years), and Google Scholar (http://scholar.google.com/; all years) were searched in June 2009 using combinations of the following search words: Mojave, Sonoran, disturbance, succession, recovery, revegetation, and change. Article titles, key words, and abstracts were scanned for these search words. Reference lists within located papers also were searched for relevant citations, and a cross-reference search was conducted using Google Scholar to identify articles that cited located papers. To qualify for inclusion in the quantitative analysis, articles had to have: (1) reported on studies conducted in the Mojave or Sonoran Deserts, (2) examined plant establishment following a discrete disturbance of known age that removed plant biomass in the case of secondary succession or created a new geomorphic surface in primary succession, and (3) provided quantitative data (e.g., plant density, cover) for both disturbed areas and undisturbed controls. The second criterion excluded grazing, because grazing is a diffuse disturbance not linked to a specific date as currently practiced with burro, horses, or livestock in this region [79].

### 2.3. Data Preparation

Quantitative data from articles meeting the inclusion criteria outlined in Section 2.2 were entered into a database. Studies used several different measures for quantifying plant community characteristics. Frequency is the proportion of sampling units occupied by a species, with sampling units commonly ranging between 1 m^2^ and 0.1 ha in size. Cover is often defined as the percent of ground area occupied by vegetation, usually allowing overlap of different species to be counted separately, such as if small plants are growing below larger plants. Density is the number of plant individuals per unit area, which I standardized for analysis to be on a per hectare basis.

There were two general types of studies in terms of the data they provided. Some studies provided only a total community measure (e.g., total plant cover, not broken down by species), and others provided community data by species, which also results in providing the total community measures. Studies that provided data by species supported analyses of the effects of disturbance on species composition (the species present and their relative abundance). After updating species nomenclature to the PLANTS Database [80], I calculated a relative measure of abundance from the data for each species in each sampling unit of these studies. Studies often reported more than one community measure (e.g., cover, density) and I used cover whenever available to calculate relative abundance. I chose cover because most studies (68%) reported cover and cover is important for ecosystem functions such as sheathing soil to limit wind erosion [11]. When cover was not available, I used whatever measure the authors provided. I computed the relative abundance measure as the proportion of the total abundance of all species provided by a given species, converted to a percentage summing to 100% on a sampling unit basis. For example, if total community cover was 20% and species A had a cover of 2%, the relative cover of species A is 10%. This relativization procedure allows species composition to be isolated from total community abundance [81], a standardization especially useful in this analysis because studies used different measures of abundance and even the same measure (e.g., frequency with different sampling unit sizes) could be measured differently by different investigators. I calculated the relative measure separately for annual and perennial plant groups, as classified by the PLANTS Database [80]. Any species with potential to be longer lived than annual was classified into the perennial group (some species can function as annual-perennial depending on weather conditions).

Some studies had specific nuances to their data which I dealt with prior to analysis. Callison *et al*. [51] provided cover of a grass that was seeded on the disturbance, and I did not include the species in this analysis. Webb *et al*. [20] combined several perennial grasses difficult to identify to species into a “grass” category, which I included in analyses of total community cover but deleted from analyses of species composition. Also for Webb *et al*. [20], different sampling units were used to measure cover and density by species, and so in using cover in this analysis, I added a nominal 0.01% raw cover to species occurring in the density (but not cover) sampling units to capture site-level species composition. I averaged data from multiple sites of the same TSD in Minnich’s [55] study. To save journal space, some studies (fewer than 15%) grouped the least abundant species into an “other species” category. I did not use these studies in the analysis of species richness, but I retained them in analyses of species composition because the “other” category typically comprised less than 5% of relative abundance.

### 2.4. Data Analysis

Addressing the study questions required using different subsets of studies and parts of the data set suitable for each question. To assess relationships of plant cover and richness with TSD, I expressed measures for disturbed areas as a percentage of undisturbed controls for each study. I then used linear regression to relate the relativized measures to TSD since linear equations typically provided as high or higher r^2^ values as non-linear equations. For species composition, I related the Sørensen community similarity index, calculated in PC-ORD software [82] as the average percent similarity of disturbed to undisturbed areas in each study, to TSD. I calculated these regressions both within studies that examined multiple TSDs (either through chronosequence or permanent plot sampling) and among studies that reported on only one TSD. Chronosequences are space for time substitutions, where different aged disturbances are sampled. With permanent plots, the same disturbance is measured repeatedly through time. To compare species composition among studies, I averaged post-disturbance species composition on a study basis and used non-metric multidimensional scaling (NMS; autopilot thorough setting) ordination in PC-ORD [82]. To compare individual species, I calculated a mean disturbed:undisturbed ratio (based on relative abundance). I also used two- (disturbed and undisturbed, not differentiated by desert) and four-category (disturbed Mojave, undisturbed Mojave, disturbed Sonoran, undisturbed Sonoran) indicator species analysis with the relative abundance of species as the data. Indicator species analysis combines the relative abundance and frequency of a species within a group to produce an indicator value that ranges from zero (no fidelity to a group) to 100 (maximum fidelity [83]). Species with indicator values ≥ 50 are considered strong indicator species [81]. For species with values ≥ 50, significance of indicator values at *P* < 0.05 was assessed using a Monte Carlo test with 1,000 permutations.

## 3. Results

### 3.1. Literature Description

The systematic search procedure uncovered 43 studies of secondary succession that met inclusion criteria for the quantitative review (Appendix). Publication dates ranged from 1961 to the present. The Mojave Desert housed 74% of the studies and the Sonoran Desert 26%. Fire was the disturbance type examined in 31% of the Mojave studies and 64% of the Sonoran studies, with other disturbances studied including transmission lines (powerline, natural gas), abandoned roads and agricultural fields, and other cleared areas such as ghost towns and military sites. Data on both annual and perennial plants were reported in 33% of the studies, annuals only in 5%, and perennials only in 63%. Recovery patterns with TSD, where three or more TSD repeated measurements were included, were assessed in 33% of the studies for at least one recovery measure, using chronosequence or permanent plot sampling methods. Results for perennial plants for secondary succession are presented first, followed by annual plants and then for four studies of primary succession.

### 3.2. Time since Disturbance Relationships

**Figure 3.**
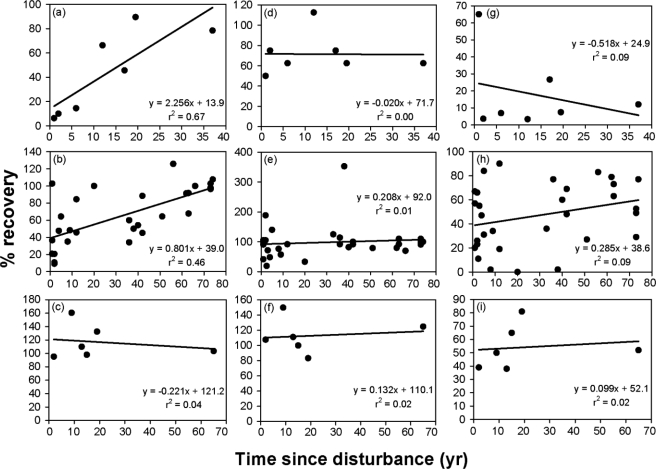
Examples of studies that examined time since disturbance (TSD) relationships with variables of plant recovery in the Mojave and Sonoran Deserts of the American Southwest. Plant cover and richness are expressed as the percent of levels found on undisturbed areas ([disturbed/undisturbed] × 100). **(a)** Perennial plant cover [51]. **(b)** Perennial plant cover using data from 29 individual studies that each made one TSD measurement. **(c)** Annual plant cover [67]. **(d)** Perennial species richness [51]. **(e)** Perennial species richness using data from 30 individual studies that each made one TSD measurement. **(f)** Annual plant richness [67]. **(g)** Disturbed:undisturbed similarity of perennial species composition [51]. **(h)** Disturbed:undisturbed similarity of perennial species composition using data from 31 individual studies that each made one TSD measurement. **(i)** Disturbed:undisturbed similarity of annual species composition [67].

In analyzing the reestablishment of perennial plant cover irrespective of the species providing the cover, cover exhibited correlations (r^2^) between 0.07 and 0.99 (mean = 0.60 ± 0.32 standard deviation) with TSD among studies, with 8 of 12 studies (67%) having correlations > 0.50 (Table 1, Figure 3). Estimates of reestablishment of cover on disturbed areas to 100% of the cover on undisturbed areas ranged from one study that found divergence with time (Hessing and Johnson [70]) to 334 years (Scoles-Sciulla and DeFalco [66]). Excepting Hessing and Johnson [70], 6 of the 11 studies (55%) had estimated reestablishment times of ≤ 41 years, and nine of 11 (82%) had estimates of ≤ 88 years (mean = 70 ± 92 years). In comparing disturbance types (fire versus land clearing), TSD correlations were generally higher for fire studies: 6 of 7 (86%) fire studies had r^2^ values > 0.66, while 0.66 was the maximum value of the five studies examining clearing disturbances. The maximum estimate for reestablishment of total cover after fire was 65 years, whereas in three of five non-fire studies recovery times exceeded 88 years. In those two remaining studies, however, cover reestablished rapidly within five years (Johnson *et al*. [65]) or exhibited fast initial recovery followed by divergence from levels found on undisturbed areas (Hessing and Johnson [67]). Data combined from 29 studies (seven of which examined fires) that each measured only one particular year after disturbance revealed an r^2^ of 0.46 with TSD and a recovery time within the range of individual studies making repeated temporal measurements after disturbance.

Species richness exhibited a different pattern with TSD than cover (Table 2, Figure 3). Richness showed weak relationships with TSD, with 8 of 10 studies displaying r^2^ values < 0.22. Those eight studies also had *y* intercepts equaling ≥ 64% recovery, indicating that substantial recovery had already occurred by the initial TSD measurements made by studies. These findings were supported using combined data from 30 studies (6 fire, 24 other), each with one temporal measurement, where the r^2^ was only 0.01 and the *y* intercept was within 8% of full recovery.

The relationship of the similarity of species composition of disturbed to undisturbed areas with TSD was mixed among studies (Table 3, Figure 3). Five of 12 studies (42%) showed r^2^ values > 0.52, while the remaining seven studies all were ≤0.30. Similarly, time to full recovery ranged from divergence (where composition of disturbed areas became less similar to undisturbed areas over time) in four studies to approximately 600 years to recovery in Vamstad and Rotenberry [67] and Webb *et al*. [63]. Combined data from 31 studies (seven of which examined fire) also showed a weak correlation of similarity with TSD and an estimated full recovery time of over 200 years.

### 3.3. Community and Disturbance Type Comparisons

Only four studies, all in the Mojave Desert, directly compared post-disturbance changes among plant community types. Vasek *et al*. [45] compared recovery along a power transmission corridor as it passed through creosote scrub and saltbush dry lake flats. In the saltbrush community, fourwing saltbush (*Atriplex canescens*), cattle saltbush (*Atriplex polycarpa*), and Mojave seablite (*Suaeda moquinii*) dominated undisturbed areas and also had recolonized 1- and 36-year-old transmission lines. Big saltbush (*Atriplex lentiformis*) was not recorded in undisturbed vegetation but comprised 17–24% of the relative cover in the disturbed areas. Different species, such as creosote bush, white bursage (*Ambrosia dumosa*), and cheesebush (*Hymenoclea salsola*), colonized the line within the creosote scrub community. Carpenter *et al*. [31] compared succession on agricultural fields abandoned 52–79 years earlier among four elevation belts (ranging from 1,100 m creosote scrub to 1,615 m sagebrush-juniper [*Artemisia tridentata*-*Juniperus osteosperma*]) in the central Mojave Desert. Middle elevations with Joshua tree woodland had 20–30% fewer species on disturbed than undisturbed areas, while the low- and high-elevation communities showed little difference in species richness with disturbance status. There was less difference in cover between disturbed and undisturbed areas in the low-elevation communities (1–3% absolute difference in cover) than in the high-elevation communities (5–8% difference). The species composition similarity of disturbed areas among the four community types ranged from 18% (1,100 m creosote scrub versus 1,615 m sagebrush-juniper) to 70% (1,280 m versus 1,430 m, both Joshua tree woodland community types). There was little difference in disturbed: undisturbed similarity among community types, as similarities ranged from 71% in creosote scrub to 85–86% in the Joshua tree woodland communities. At Yucca Mountain, Nevada, Gabbert *et al*. [54] compared species composition among four community types disturbed by clearing with heavy equipment 6–12 years earlier. Disturbed creosote-bursage communities were more than twice as similar (61% Sørensen similarity) to their paired undisturbed areas as blackbrush (22%), boxthorn-hopsage (*Lycium andersonii*-*Grayia spinosa*; 28%), or mixed species communities (26%).

Minnich [55] was the only study that directly compared TSD effects among community types. He used a chronosequence of 6–20 years TSD in blackbrush and 1–47 years in Joshua tree woodland to examine post-fire recovery in Joshua Tree National Park in California. Regression equations of the % reestablishment of cover with TSD were similar for the two community types: *y* = 2.426TSD + 49.2, *r* = 0.77, 21 years to 100% recovery (blackbrush), and *y* = 1.832TSD + 41.4, *r* = 0.84, 32 years to 100% recovery (Joshua tree woodland). Disturbed:undisturbed similarity was 62% on a 20-year-old blackbrush burn and 69% on a 21-year-old Joshua tree woodland burn.

In comparing disturbance types, based on the TSD relationships presented in section 3.2, cover reestablished more rapidly overall after fire than other types of disturbance involving land clearing (Table 1). Results for recovery of species richness and species composition were mixed (Tables 2, 3). Within land-clearing disturbances, several authors have noted the possibility that the intensity of disturbance (e.g., amount of surface soil removed) or the degree of soil compaction influences recovery. For example, by studying abandoned roads in the eastern Mojave Desert, Bolling and Walker [27] found that road type (either track roads created simply by driving, or bladed roads created by bulldozing a path) influenced some soil properties, but its effects on plant establishment were not fully clear. At 56-year-old military disturbances in the western Sonoran Desert in Arizona, Kade and Warren [8] found that plant cover was six-fold greater on former vehicle staging areas than in former tent areas, even though the degree of soil compaction did not differ significantly between the areas. In a different abandoned military training area (40 years old at the time of the study), in the eastern Mojave Desert, Prose *et al*. [52] reported that cover was lowest on roads compared to tent areas and parking areas. Roads overall had the most compacted soils. Similarly, Webb and Wilshire [48] found that cover was three times lower on a former road than housing areas at the ghost town site of Wahmonie in the northern Mojave Desert. While compaction was greatest on the road, TSD differed as the road was abandoned 18 years earlier and the housing areas 51 years earlier. Based on examining a chronosequence of a variety of ghost town disturbances varying in age from 19–92 years, Webb and Thomas [62] concluded that effects of soil compaction on vegetation recovery could not be discerned due to high variability. Examining the amount of soil removed by disturbance, Vasek [24] noted that succession was slower on the bottoms of a pit (where more soil had been removed) than on pit sides at a California site in the Mojave Desert.

### 3.4. Among-Study Comparisons

**Figure 4.**
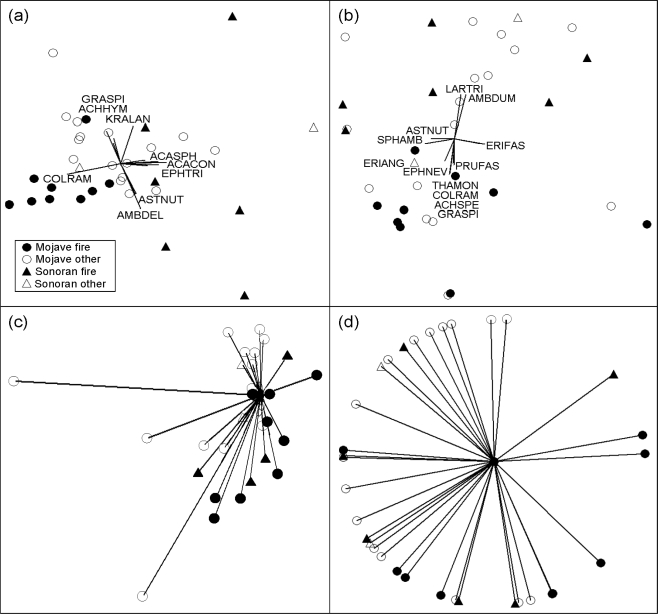
Illustration of species composition patterns among studies examining post-disturbance plant recovery in the Mojave and Sonoran Deserts of the American Southwest. Studies are distinguished by desert and disturbance type (fire or land-clearing disturbances such as old roads). **(a)** Undisturbed vegetation, where species are shown as vectors. Vector lengths and directions indicate correlations with different study groupings. Only species exhibiting an r^2^ ≥ 0.15 are shown. Species are abbreviated as the first three letters of the genus and species given in Table 4, and for four species in (a) and (b) not given in Table 4, as ACACON = *Acacia constricta*, ASTNUT = *Astragalus nuttallianus*, EPHTRI = *Ephedra trifurca*, and ERIANG = *Eriodictyon angustifolium*. **(b)** Disturbed vegetation. **(c)** Successional vectors showing the difference between disturbed and undisturbed species composition of each study, where increasing lengths of vectors represent greater differences. **(d)** Successional vectors standardized to unit length, comparing the direction of species compositional change among studies.

Ordinations allowed patterns of average disturbed and undisturbed community composition to be compared among studies. There was some separation of undisturbed vegetation by desert, with Sonoran studies generally located on the right side of the ordination, but some intermingling occurred with Mojave studies in the center of the ordination (Figure 4a). Studies in the Sonoran uplands (those containing saguaro cacti and mixed shrub-trees) were the best separated from Mojave studies, whereas Sonoran studies in creosote scrub were less distinct from Mojave studies.

Disturbance was associated with a loosening of the groupings (Figure 4b). For instance, Mojave Desert undisturbed vegetation for fire studies was tightly grouped, whereas burned plots were dispersed and intermingled with other disturbance types. Deserts still separated, with Sonoran studies in the upper part of the ordination, but intermingling occurred with Mojave studies in the ordination center as with undisturbed vegetation. Community groupings also remained recognizable based on study groupings and species correlation vectors, as creosote scrubland grouped in the upper part of the ordination, blackbrush shrubland on the bottom, and mixed species communities on the left.

There were no clear patterns in the lengths of successional vectors, indicative of the relative amount of difference in species composition between disturbed and undisturbed areas, among studies of different disturbance type or between deserts (Figure 4c). However, the three longest vectors, suggesting the greatest amount of difference between disturbed and undisturbed vegetation, all originated from studies of non-fire disturbances. There also was considerable overlap in the direction of vectors, indicative of successional trajectories, between disturbance type and deserts (Figure 4d).

### 3.5. Individual Perennial Species

Occurrences of individual species were diffuse (*i.e.*, many absences) across the diversity of studies, and as a result, indicator species analysis did not identify any species (out of 199 species recorded across studies) that had indicator values >49 for disturbed/undisturbed categories by desert. This is below the cutoff of 50 to be considered strong indicator species. However, the most abundant species could be classified into the general response types to disturbance as increasers, versatile (species important in both disturbed and undisturbed habitats), and decreasers (Table 4). Increasers exhibited a high disturbed:undisturbed abundance ratio averaged across studies and also had high abundance when present in a study in disturbed areas relative to undisturbed areas. This measure of abundance when present was important to examine because, owing to the diversity of studies and sites, few species occurred in more than half of the studies. The most common species included creosote bush (occurring in 48% of disturbed studies and 64% of undisturbed studies), Nevada jointfir (*Ephedra nevadensis*; 45% disturbed, 52% undisturbed), cheesebush (52% disturbed, 42% undisturbed), and white bursage (45% disturbed, 42% undisturbed).

Major increasers included forbs and shrubs such as brittlebush (*Encelia* spp.), desert trumpet (*Eriogonum inflatum*), whitemargin sandmat (*Chamaesyce albomarginata*), broom snakeweed (*Gutierrezia sarothrae*), rubber rabbitbrush (*Ericameria nauseosa*), desert globemallow (*Sphaeralcea ambigua*), and brownplume wirelettuce (*Stephanomeria pauciflora*). Two grasses—Indian ricegrass (*Achnatherum hymenoides*) and desert needlegrass (*Achnatherum speciosum*)—also were prominent increasers, especially considering that the species were among the most frequently detected disturbance species among studies. Versatile species had disturbed:undisturbed ratios near 1, and were typified by Nevada jointfir, white bursage, and boxthorn. The grass big galleta (*Pleuraphis rigida*) also was versatile, as were banana yucca (*Yucca baccata*) and Mojave yucca (*Yucca schidigera*), at least on a relative abundance basis even if their absolute abundance declined on disturbed compared to undisturbed areas. Blackbrush exemplified decreasing species and exhibited the largest absolute decline after disturbance when present in a study. Triangle bursage (*Ambrosia deltoidea*) and creosote bush also incurred major reductions after disturbance when present, but these species still maintained relative abundances >11% in disturbed areas. Other decreasers included Joshua tree and various cacti (e.g., pencil cholla [*Cylindropuntia ramosissima*], Engelmann's hedgehog cactus [*Echinocereus engelmannii*]), and hopsage.

### 3.6. Annual Vegetation

Annual plant cover rebounded rapidly after disturbance, with two of four TSD studies showing *y*-intercepts reflecting cover greater in disturbed than undisturbed areas (Table 5, Figure 3). The other two studies showed reestablishment to amounts found on undisturbed areas in two years or less. Species richness exhibited a similar trend, as two studies had intercepts near 100%, and the other three studies had estimated recoveries of ≤13 years. For disturbed and undisturbed community similarity, intercepts ranged from 50–92%, indicating high similarity, though completing the rest of recovery was estimated to require much longer.

It was difficult to make quantitative comparisons of annual plant species composition and individual species among studies because fewer studies examined annuals than perennials. However, some trends appeared from the collective results of individual studies. First, several studies reported a strong temporal influence (linked to precipitation) on the reestablishment of annual plants, where rainfall amounts in a given year could overwhelm any effects of disturbance (e.g., [53,60,76]). In some years of low rainfall virtually no annuals may be recorded, whereas in moist years, biomass and cover may increase several orders of magnitude. Second, Brooks [60] and Cave and Patten [72] highlighted the effects of microsite (e.g., below shrubs versus in interspaces between shrubs) on the distribution and abundance of annual plants in both disturbed and undisturbed areas. Overall, annual plants were more abundant below shrubs in both areas, but some annual species showed contrasting patterns.

Third, different annual species have been reported to show different responses to disturbances. For example, the non-native grass red brome has often decreased the first few years following disturbance in permanent plot studies [53,60,72]. Redstem stork's bill (*Erodium cicutarium*), in contrast, has increased rapidly [53,57,60,65,73]. Native annuals reported to colonize disturbed areas include desert Indianwheat (*Plantago ovata*) following fire and land clearing in both the Mojave and Sonoran Deserts [64,72,76], whitestem blazingstar (*Mentzelia albicaulis*) after nuclear detonation tests in the northern Mojave Desert [42], flatcrown buckwheat (*Eriogonum deflexum*) on abandoned roads and cleared areas in the Mojave Desert [57,64], and species such as cryptantha (*Cryptantha* spp.) and sandmat (*Chamaesyce* spp.) in various studies. Fourth, studies making fewer than three temporal measurements of disturbed:undisturbed community composition similarity typically supported the TSD studies in showing high similarity between disturbed and undisturbed areas. For example, Brown and Minnich [73] found that community composition was 73% similar between burned and unburned areas 3–5 years after fire in the upper Sonoran Desert. Prose and Wilshire [28] reported that similarity ranged between 73 and 84% 22 and 43 years after military disturbances in the eastern Mojave Desert.

### 3.7. Primary Succession

Four studies of primary succession that met selection criteria were uncovered, each of which examined different geomorphic surfaces varying in age [20,33,77,78]. Specifically, these studies assessed debris flows (also termed alluvial fans), resulting from the transport and deposition of soil material at the mouths of canyons and below mountains. Relative ages of these flows can be estimated by known dates of flood events for recent flows and the degree of soil development for older flows [33,78]. Ages of flows examined by these four studies ranged from 5 years to tens of thousands of years. All of the studies reported on perennial plants; none reported on annuals.

Bowers *et al*. [33] examined debris flows ranging in age from 5 to 3,100 years along the Colorado River in Grand Canyon National Park in Mojave-Sonoran transitional ecosystems. In using the 3,100-year-old flow as the undisturbed data and flows 5 to 485 years old as the successional sites, the relationship between TSD and cover reestablishment was: % of undisturbed = 0.1476TSD + 73.8 (*r* = 0.69). For species richness, all nine TSDs except for two exceeded 100% of richness of undisturbed areas, resulting in a *y* intercept of 152%. There was a close correspondence between TSD and the Sørensen similarity with undisturbed areas: similarity = 0.052TSD + 3.4 (*r* = 0.88). These authors noted that there was a relatively orderly progression where short-lived species (e.g., broom snakeweed, brittlebush, brownplume wirelettuce) dominated the young flows and longer lived species like creosote bush (which was not detected on flows < 285 years in age) inhabited the older flows.

In Death Valley National Park in the western Mojave Desert of California, Webb *et al*. [20,30] reported that a young debris flow (age 5 years) in Wood Canyon was occupied by grape soda lupine (*Lupinus excubitus*) and threadleaf snakeweed (*Gutierrezia microcephala*), species not detected on older surfaces. In contrast, blackbrush cover increased from 0.2% on the young flow to 18% on the intermediate-aged flow (thousands of years old) and 21% on the oldest flow (tens of thousands of years old). Total perennial cover ranged from 4% on the young flow to 28% on the oldest flow. In a different chronosequence in Gold Valley, species such as cheesebush, narrowleaf goldenbush (*Ericameria linearifolia*), Nevada jointfir, and white bursage dominated density on the youngest (<100 years in age) flow, whereas creosote bush, white bursage, boxthorn, hopsage, and Nevada jointfir dominated the oldest (late Pleistocene) flow.

Similarly, McAuliffe [77] found that debris flows ranging in age from several centuries to several millennia supported different suites of species in the western Sonoran Desert. Creosote bush was only found on the intermediate and oldest surfaces and was most abundant on the oldest. In another study, this one in the eastern Sonoran Desert Uplands of Arizona, McAuliffe [78] also noted that abundance of creosote bush was linked to surface age and stability, but specific patterns depended on local soil characteristics like the presence of clay-rich argillic horizons.

## 4. Discussion

### 4.1. Analysis Challenges

Several aspects of synthesizing the available data may have affected results of the analysis of secondary succession studies. A first challenge in analyzing species composition data from the literature is that due to data summary techniques or journal space limitations, all species are not always reported in publications (often only major species are reported). However, since the relative abundance of these non-reported species is low (often precisely why they are chosen not to be reported), their effect on community similarity analyses such as the Sørensen index used in this analysis is minimal. For example, Minich [55] included an “other” species category in his data, but these species averaged only 6% of the total relative community cover in both burned and unburned areas. I avoided this issue of not having access to full species lists for the analysis of species richness, as I did not include any study in TSD-richness analyses that did not report actual richness or all species from which I could calculate richness. A second challenge is that different measures (e.g., cover, density) of plant abundance are reported among studies, and these measures may emphasize different aspects of the community. In this study, cover was chosen as the primary measure for analysis, as cover was reported in 68% of community studies of secondary succession and is a long-used measure in vegetation sampling [84]. Whatever measure was reported had to be used to analyze data from studies that did not report cover. Analyzing different measures, as well as the possibility that different investigators measured cover differently, was attempted to be overcome by calculating a relative abundance measure standardized across all studies to range from 0–100% for species. A further challenge in the analysis of TSD-species richness relationships is that sample areas differed among studies (richness increases with increasing area sampled) and some did not even report areas. Brooks and Matchett [61] was the only study that reported richness for different sized sample areas, and TSD-richness results did differ among two scales in that study (Table 2). This observation suggests that different scales of analysis could have resulted in some of the variation in the TSD-richness relationships. In addition, the amount of recovery after disturbance was compared in a relative manner to nearby undisturbed areas. However, as studies such as Turner *et al*. [85] illustrate, humans have long impacted vegetation in southwestern deserts. Vegetation in the “undisturbed” control areas may itself have been disturbed in ways difficult to detect, although these areas still served as comparison areas not subject to the recent intensive disturbances on disturbed areas.

The number of sites and years since disturbance in chronosequence and permanent plot studies could have affected TSD relationships, as some studies had as few as three sites or repeated measures. Finding sufficient sites and TSDs is challenging in many disturbance-related research projects. Disturbances such as wildfire are by nature unplanned events, and similar to past disturbances such as ghost towns, researchers work with what disturbances are available and have a documented history. As Webb *et al*. [86] note, recovery is not necessarily linear, which makes extrapolating from short TSDs difficult. While linear equations generally fit the data as well or better than non-linear equations for available TSD relationships (Tables 1–3), the short time periods available for analysis in some studies may not have enabled a full representation of post-disturbance recovery rates. It should be noted, however, that estimating long-term recovery was not necessarily a goal of the original studies, such as Scoles-Sciulla and DeFalco [66], whose study objective was to quantify early recovery patterns. Short-term studies that measured only 1–2 years were useful for the regressions of combined studies of overall patterns. Webb *et al*. [20,86] provide a further discussion of the challenges inherent in extrapolating recovery, such as when droughts may result in shifts in recovery patterns [87].

It was difficult to isolate overall desert, community, and disturbance type effects in the analysis, because other variables (e.g., TSD) were not necessarily constant among studies. This is to be expected since study contexts differed. Averaging post-disturbance species composition in the ordinations resulted in averaging different TSDs among studies, although this analysis provided an overall disturbed-undisturbed comparison across studies. There is a need for additional studies that try to keep as many external variables constant, such as TSD, to isolate disturbance and community type effects within their study. This is again challenging due to the unplanned and retrospective aspect of disturbances, and is probably why Minnich [55] was the only study to compare recovery of community types through time. Even in Minnich’s [55] study, however, the available TSD was 47 years for the Joshua tree community and only 20 years for the blackbrush community.

It also was difficult to isolate patterns of individual species statistically (through indicator species analysis and as vectors in the ordinations) because of the diversity of species composition across the studies that spanned different deserts, sites, and communities. Therefore, a species might have high fidelity to disturbance but only occur in a small subset of studies that were conducted in habitat suitable for the species, meaning that the species had many absences. Simple descriptive measures such as the disturbed:undisturbed abundance ratio and mean abundance when present appeared effective for an overall characterization of species responses to disturbance (Table 4).

### 4.2. Evidence for Desert Succession Concepts

Data gathered by the synthesis can be used to assess evidence concerning several concepts about succession in arid lands advanced in the literature. Succession was purported by some early authors not to actually occur in deserts [21,22]. The four studies of primary succession (Appendix) provide the strongest evidence that succession can occur in deserts, as these studies illuminated a long-term progression of plant community development perceived using the chronosequence approach. Studies of secondary succession illustrated that there are multiple stages of colonizing communities after disturbance, and four studies estimated that succession resulted in the reestablishment of communities typical of undisturbed areas in 9–33 years (Table 3). However, other studies reported that disturbed communities continued to diverge from undisturbed communities with increasing TSD, or estimated recovery times for species composition of >500 years [63,67]. Divergence could imply that succession was not occurring, change was proceeding to a different community or set of communities than nearby undisturbed areas, or methodological challenges (e.g., durations of studies, limitations of the chronosequence approach) made estimating successional progression and time scales difficult.

Several authors have asserted that during succession in deserts, perennial community composition generally progresses from short- to long-lived species (e.g., [32,33]). Data on the life spans of individuals are required to assess this concept, such as the life-span data in Bowers *et al*. [33]. This postulate appears supported by the data, as several species reported to increase after disturbance (Table 4) are considered short lived. For example, the early colonizers sweetbush (*Bebbia juncea*), broom snakeweed, and brownplume wirelettuce are reported to live only 20 years, rather than the hundreds and thousands of years reported for some late-successional species like honey mesquite (*Prosopis glandulosa*) and creosote bush [33]. Life-history data are not available for many of the other species, and such information could be useful for refining our understanding of the traits typifying early versus later colonizers.

Annuals have been purported to be major components of both young and old communities in deserts [32]. This synthesis found strong evidence supporting this supposition, as annual plant cover and richness rebounded rapidly after disturbance to levels similar to or exceeding old, undisturbed communities (Table 5). In the three studies assessing recovery of annual species composition, *y* intercepts exceeded 50% similarity with undisturbed communities, also implying rapid recovery. Data from studies of old desert communities also suggest that annuals are important in old communities in moist years [53,61,72]. The prevalence of annuals in young and old communities in deserts differs from common views of succession in temperate regions, where annuals are considered to be prominent mainly during early succession [5].

Another concept advanced in the literature is that many of the post-disturbance increasing species also inhabit washes, which are disturbed naturally by periodic floods [23]. To evaluate evidence for this, I compared the early successional species in Table 4 with studies of desert wash species composition (e.g., [88]). Some of the early colonizers in Table 4 have been reported as dominants in washes; for example, cheesebush, brittlebush, and desert almond (*Prunus fasciculata*). Reciprocally, many species dominating washes, such as honey mesquite, have not been reported as major early colonizers of disturbances. These species may have water and other habitat requirements that are met in washes but not in upland ecosystems, independent of disturbance.

Contingency effects, in particular disturbance type, severity, and community type, have been suggested to affect recovery rates and the course of succession (e.g., [30]). Overall it appeared that recovery after fire versus land-clearing disturbances did differ, with perennial cover generally rebounding faster after fire compared to other disturbances (Table 1). Post-disturbance species composition also differed between fire and land-clearing disturbance types based on the ordinations (Figure 4). Although fire influences soil physical and chemical properties [56], soils may still remain more intact after fire than land-clearing disturbances where they are scraped away or heavily compacted. Furthermore, unlike after land-clearing disturbances, roots and seeds are not necessarily entirely removed by fire. These residual propagules may enhance plant reestablishment on fires relative to other disturbance types [89].

Within land-clearing disturbances, definitive conclusions about the effects of factors such as the amount of soil removed or the degree of soil compaction on recovery cannot be made from the available data. Studies produced mixed results, or high variability precluded detecting any potential trends (e.g., [27,62]). It appeared that due to vehicle traffic, abandoned roads had more soil compaction than many other disturbance types, which may hinder plant recovery [48,52]. On the other hand, the narrow, linear configuration of roads may facilitate seed dispersal from surrounding undisturbed areas for recolonization readily compared to larger disturbances further from seed sources [90]. This example illustrates the difficulties in isolating effects of disturbance type and severity from other confounding variables in the retrospective study of non-experimental disturbances. Understanding effects of factors such as the amount of soil removal and compaction on plant recovery is important for making management decisions when revegetation is a goal. For instance, it is important to know whether the benefits outweigh the expense of ameliorating soil compaction through ripping with heavy equipment or salvaging soil to re-apply after disturbance.

In comparing succession among community types, Carpenter *et al*.’s [31] study of old fields in the Mojave Desert indicated that post-disturbance species composition differed among four communities. There was little difference, however, among community types in the similarity of each to their own undisturbed control. In contrast, Gabbert *et al*. [54] found that creosote-bursage communities in the northern Mojave Desert were more than twice as similar to paired undisturbed areas as blackbrush and two other community types, implying that the creosote communities had recovered the fastest. More work is needed to understand how succession may differ among community and soil types [59]. Differences could hinge on several factors, such as the elevation of the community affecting precipitation, soil moisture and rooting depth, and local genetic adaptations and traits of the species in the community [31,36,91]. For example, blackbrush does not resprout after disturbance, whereas creosote bush has some sprouting ability, potentially hastening its reestablishment [15].

Several other contingency effects could have major influences on post-disturbance succession. For example, rainfall amounts in years following a disturbance could affect the type of vegetation that initially establishes, which in turn could influence subsequent community development [13,63]. The differences in seasonality of precipitation, where rainfall is more bimodal or evenly distributed in the Sonoran compared to the Mojave Desert [36], between deserts also could influence post-disturbance establishment of both annual and perennial species [38]. Grazing by herbivores such as wild burros and jackrabbits is another example of a contingency effect that could filter which species are able to inhabit the post-disturbance environment and could influence the total cover of post-disturbance vegetation [79].

### 4.3. Conservation and Management Implications

If a management objective is to maintain old perennial plant communities such as those containing Joshua tree, giant saguaro, creosote bush, and blackbrush, probably the best strategy is to avoid disturbing these communities in the first place. Strategies to accomplish this could include minimizing unauthorized off-road driving through the desert, limiting unnecessary land clearing, reducing damage by non-native animals such as burros, and actively suppressing and reducing wildfires [1,79,92]. These strategies will not always be possible, such as for fire suppression where fuel-producing non-native annual grasses have invaded even relatively undisturbed desert, challenging fire suppression efforts across large landscapes [93]. When old plant communities are disturbed, the literature suggests that recovery times for species composition are on the order of decades to centuries at a minimum (Table 3). In fact, examples of long-term community composition establishment must largely be derived from studies of long primary successions, as typically only initial regeneration trends were able to be captured in the <100 year time frames available for studies of secondary succession. Furthermore, as Webb *et al*. [20] note, climate and ecological conditions (e.g., relatively recent establishment of non-native species) have changed from the conditions in which many of the old communities became established. This further complicates making recovery estimates.

Land managers can expect, however, that colonization by early successional communities will facilitate the reestablishment of total perennial cover (to amounts found on undisturbed areas) generally within 100 years, and in fewer than 40 years in some situations (Table 1). These early successional communities may provide habitat favorable for some wildlife species. For instance, Simons [94] found that Merriam’s kangaroo rat (*Dipodomys merriami*), a species that forages in open areas, increased after fire in Sonoran Desert upland habitat. Additionally, annual communities seem to reestablish fairly rapidly, and in moister years little difference may exist in cover and species composition between disturbed and undisturbed areas after some disturbances (Table 5). In fact, total annual cover may actually increase following disturbance, although it is important to differentiate responses of non-native versus native species. Early successional communities of both perennial and annual species can be quite diverse given the commonly observed rapid reestablishment of species richness, which could promote landscape heterogeneity. Further research would be useful to determine the habitat and resource value (e.g., habitat for wildlife, carbon sequestration) of different aged desert communities, as this has not been well studied.

Understanding natural recovery patterns may be valuable for informing revegetation treatments if decisions are made to actively revegetate disturbed areas by either augmenting establishment of early colonizers, reintroducing late-successional species, or both. Early successional species apparently do not require the below-shrub fertile island microsites to become established that are critical for the establishment of many late-successional species like creosote bush [95]. It is not understood, however, whether creating fertile islands also could enhance establishment of early colonizers. Further studies such as Carrillo-Garcia [96] and Butterfield and Briggs [97] that examine the formation of fertile islands and plant transitions on these microsite scales may be helpful for determining if creating fertile islands would hasten succession [98,99]. Likewise, augmenting establishment of early successional species may help establishment of later colonizers, if the facilitation model of succession of moist regions applies to deserts [100]. If facilitation occurs, the early colonizers would ameliorate the post-disturbance environment, making it more habitable for later colonizers. For actively revegetating with late-successional species, a recent review of revegetation practices in the Mojave Desert concluded that seeding and planting are prone to failure but can be successful in some instances [18]. Success can hinge upon many factors including species selection, genetics of the plant stock, climate, short-term weather after revegetation, the severity of the disturbance to be revegetated, and the revegetation technique itself (e.g., seeding versus directly planting greenhouse-grown seedlings). For example, creosote bush was not observed naturally colonizing new surfaces (which would have to be via seed) in primary succession for at least several hundred years, supporting the observation that creosote rarely establishes by seed [33,77]. Consistent with these natural patterns, directly planting creosote seedlings in active revegetation projects was more effective than seeding [18]. Planting enabled the rarely successful seed germination and vulnerable early seedling establishment phases to be bypassed. This type of knowledge, informed by studying patterns of natural post-disturbance recovery, may be crucial to the success of revegetation and restoration projects.

### 4.4. Summary and Conclusion

A quantitative review uncovered 47 studies that have evaluated post-disturbance plant recovery and succession in the Mojave and Sonoran Deserts of the American Southwest. Succession seems to occur in deserts but transpires over a longer time period than in more temperate regions. After disturbances such as fire that do not physically remove or heavily compact soils, perennial plant cover in these deserts can rebound, in some instances, to levels similar to undisturbed areas within 40 years. Species richness, although derived from different actual species on disturbed versus undisturbed areas, often reestablishes more rapidly than cover owing to an influx of new species arriving after disturbance. Both species richness and cover rebound more rapidly than species composition. Annual plant communities appear to recover more rapidly than perennial vegetation, and may even exhibit greater post-disturbance cover on disturbed than undisturbed areas. It is important to distinguish responses of native and non-native species, however. The observation of rapid annual recovery, together with the observation that many of the early colonizing perennials generally have short life spans, support the supposition that communities generally shift from short- to long-lived species as succession proceeds. This is not unlike the concept of succession for temperate regions, although in deserts annuals remain abundant in old communities during wet years. Versatile desert species, however, such as white bursage and Nevada jointfir, may be long lived and inhabit both young and old communities. For some species, their versatility may result primarily from establishment by seed (e.g., white bursage) or sprouting (e.g., Mojave yucca). As in temperate regions, deserts contain species that are benefited or reduced by disturbance.

Future research on desert succession could refine our understanding of differences among disturbance and community types and how much time is required for recovery of different vegetation characteristics. Most research has been constrained to studying existing disturbances, which has often made it difficult to directly compare disturbance and community types because variation in other variables confounds these comparisons. Experimental research together with revisiting studies of existing disturbances to generate longer term records of succession could advance knowledge of specific variables influencing succession. These contingency effects, including climate, the presence or absence of non-native species, or the occurrence of multiple disturbances, could have major influences on succession but are poorly understood. The traits (e.g., growth rate, seed germination, nutrient requirements) of early colonizers also are not well understood, and this type of information could be useful for identifying species most amenable to different active revegetation treatments. Similarly, processes, such as the formation of fertile islands important for establishment of some species, could have major influences on succession but are not well known [97]. Especially given the large land area in deserts already occupied by burns, we need a better understanding of the function provided by different aged burns. For instance, future research could examine how carbon storage capability and suitability of wildlife habitat could change with increasing time since disturbance. While successional communities may have unique values, the data suggest that protecting deserts from disturbance (e.g., unauthorized off-road driving, fire) is critical for sustaining old communities. Since disturbances can leave scars in the desert visible for multiple human generations, great care should be exercised before disturbing the desert, including with human-caused fire ignitions.

This synthesis can help us understand the consequences of disturbance and recovery of land and how they influence public health and well being. Disturbing the desert has a variety of effects on human habitations and structures (e.g., wildfires that threaten homes, soil erosion that can damage roads), biodiversity including genetic material, soil and plant productivity (influencing capability for agriculture, ranching, and other land uses), natural resource availability, economics (e.g., tourism industry and scenic landscapes), aesthetics, carbon sequestration, air quality (e.g., generation of hazardous dust on de-vegetated landscapes), and many other features that directly affect public health [1,14,17]. An additional, emerging public health consideration in deserts is the potential for the establishment of broad-scale alternative energy projects [101]. Owing to their abundant open land and sunshine, deserts are considered good candidate locations for technologies such as solar energy. For example, hundreds of square kilometers of public land held by the US Bureau of Land Management are under consideration for solar energy projects in the Mojave Desert and surrounding arid lands. As currently envisioned, these projects involve blading the soil and clearing vegetation for constructing solar structures and support facilities [102]. While alternative energy can be viewed as providing extensive public benefits, ironically environmental damage from these projects can be severe [101]. These projects, for example, can impact aesthetics, consume large amounts of resources such as water, limit other land uses, preclude public access to public land, and destroy habitat for endangered species. Unfortunately, this synthesis found that no studies have evaluated disturbances associated with alternative energy in these deserts. Given the broad-scales of disturbance proposed by these projects, it is important to understand how damage to natural resources can be minimized and how ecosystems can recover from short-term (e.g., temporary roads associated with construction of facilities) and long-term (e.g., off-site impacts) disturbances connected with energy installations. The long recovery estimates associated with some of the land-clearing disturbances documented in this review may be applicable for how long plant communities require for recovery following similar disturbances linked with energy development. However, this review suggests that specific disturbances resulting from alternative energy projects have not been studied at all. Documented information is urgently needed for supporting project planning to assess the impacts of these disturbances, how the disturbances can be minimized, and how recovery from the disturbances can be promoted.

## Figures and Tables

**Table 1.** Relationship of time since disturbance (TSD) and perennial plant cover in the Mojave and Sonoran Deserts of the American Southwest.

Reference	Disturbance type	TSD (yr)	Sampling Method^1^	No. yrs.^1^	Cover^2^ = mTSD + b			Yrs. to 100%^3^
					m	b	*r*	
Johnson *et al.* [68]	Powerline corridor	1–6	PP	6	16.294	20.5	0.42	5
Hessing and Johnson [70]	Powerline corridor	1–5	PP	5	−7.372	88.1	–0.81	—
Callison *et al.* [51]	Fire	1–37	CS	7	2.256	13.9	0.82	38
Medica *et al.* [53]	Fire	2–8	PP	3	4.522	–7.0	0.95	24
Minnich [55]	Fire	1–47	CS	9	1.854	44.5	0.82	30
Bolling and Walker [27]	Abandoned road	5–88	CS	7	0.457	33.4	0.49	146
Brooks and Matchett [61]	Fire	6–14	CS	3	1.630	33.5	0.26	41
Webb and Thomas [62]	Ghost town	19–92	CS	24	1.130	0.0	0.66	88
Webb *et al.* [63]	Fire	4–41	PP	3	1.416	7.9	0.99	65
Alford *et al.* [29]	Fire	5–21	CS	4	2.635	21.9	0.99	30
Scoles-Sciulla and DeFalco [66]	Abandoned road	1–7	CS	4	0.299	0.0	0.73	335
Vamstad and Rotenberry [67]	Fire	2–65	CS	6	1.589	38.1	0.96	39
29 studies, 1 year of data each	Fire, other	1–74	—	—	0.801	39.0	0.68	76

1 CS = chronosequence, PP = permanent plot. No. yrs. represents how many different years were represented by the sampling.

2 Cover is expressed as a percentage of undisturbed areas.

3 Years required for cover to reach 100% of the cover on undisturbed areas, as estimated by the regression equation.

**Table 2.** Relationship of time since disturbance (TSD) and perennial plant species richness in the Mojave and Sonoran Deserts of the American Southwest.

Reference	Disturbance type	TSD (yr)	Sampling method^1^	No. yrs.^1^	Richness^2^ = mTSD + b			Yrs. to 100%^3^
					m	b	*r*	
Hessing and Johnson [70]	Powerline corridor	1–5	PP	5	1.175	75.2	0.26	21
Callison *et al.* [51]	Fire	1–37	CS	7	−0.020	71.7	0.00	—
Lei [56]	Fire	1–17	CS	4	1.068	38.6	0.46	58
Bolling and Walker [27]	Abandoned road	5–88	CS	7	0.237	63.8	0.20	152
Brooks and Matchett [61] - 0.01 ha	Fire	6–14	CS	3	0.357	74.2	0.00	72
Brooks and Matchett [61] - 0.1 ha	Fire	6–14	CS	3	−0.398	84.2	−0.33	—
Webb *et al.* [63]	Fire	4–41	PP	3	3.482	55.3	1.00	13
Scoles-Sciulla and DeFalco [66]	Abandoned road	1–7	CS	4	13.861	0.0	0.73	7
Vamstad and Rotenberry [67]	Fire	2–65	CS	6	−0.379	92.9	−0.26	—
30 studies, 1 year of data each	Fire, other	1–74	—	—	0.208	92.0	0.10	38

1 CS = chronosequence, PP = permanent plot. No. yrs. represents how many different years were represented by the sampling.

2 Richness is expressed as a percentage of undisturbed areas.

3 Years required for richness to reach 100% of the richness on undisturbed areas, as estimated by the regression equation.

**Table 3.** Relationship of time since disturbance (TSD) and community percent similarity (Sørensen index) for perennial plants in the Mojave and Sonoran Deserts of the American Southwest.

Reference	Disturbance type	TSD (yr)	Sampling method^1^	No. yrs.^1^	Similarity = mTSD + b			Yrs. to 100%^2^
					m	b	*r*	
Johnson *et al.* [68]	Powerline corridor	1–6	PP	6	10.600	7.4	0.80	9
Hessing and Johnson [70]	Powerline corridor	1–5	PP	5	−1.250	95.5	−0.55	—
Callison *et al.* [51]	Fire	1–37	CS	7	−0.518	24.9	−0.30	—
Medica *et al.* [53]	Fire	2–8	PP	3	2.833	47.2	0.97	19
Minnich [55]	Fire	1–47	CS	9	0.560	42.8	0.51	102
Lei [56]	Fire	1–17	CS	4	−0.448	23.4	−0.32	—
Bolling and Walker [27]	Abandoned road	5–88	CS	7	0.295	44.7	0.35	187
Brooks and Matchett [61]	Fire	6–14	CS	3	−0.692	12.5	−0.72	—
Webb *et al.* [63]	Fire	4–41	PP	3	0.147	14.3	0.45	582
Alford *et al.* [29]	Fire	5–21	CS	4	2.160	28.0	0.91	33
Scoles-Sciulla and DeFalco [66]	Abandoned road	1–7	CS	4	4.158	0.0	0.73	24
Vamstad and Rotenberry [67]	Fire	2–65	CS	6	0.127	25.4	0.14	587
31 studies, 1 year of data each	Fire, other	1–74	—	—	0.285	38.6	0.30	215

1 CS = chronosequence, PP = permanent plot. No. yrs. represents how many different years were represented by the sampling.

2 Years required for similarity to reach 100% between disturbed and undisturbed areas, as estimated by the regression equation.

**Table 4.** Categorization of response to disturbance for major perennial species in the Mojave and Sonoran Deserts of the American Southwest.

Species	R^1^	AD	AU	OD	OU	ADP	AUP
Increasers		———— % ————					
*Acamptopappus sphaerocephalus*	1.6	1.4	0.9	12	18	11.4	4.8
*Achnatherum hymenoides*	4.3	1.2	0.3	21	15	5.7	1.9
*Achnatherum speciosum*	7.4	5.9	0.8	30	33	19.5	2.4
*Baileya multiradiata*	2.9	1.3	0.5	18	12	7.2	3.7
*Bebbia juncea*	4.9	0.2	0.0	9	12	2.6	0.4
*Chamaesyce albomarginata*	36.5	1.2	0.0	9	6	12.8	0.5
*Encelia farinosa*	1.6	1.5	0.9	21	15	7.1	6.2
*Encelia frutescens*	84.2	1.4	0.0	9	6	15.9	0.3
*Encelia virginensis*	5.1	1.6	0.3	21	18	7.7	1.8
*Eriogonum inflatum*	85.0	0.2	0.0	9	3	2.1	0.1
*Ericameria nauseosa*	237.6	0.5	0.0	15	3	3.5	0.1
*Gutierrezia sarothrae*	7.6	2.6	0.3	18	21	14.6	1.6
*Hymenoclea salsola*	2.1	7.0	3.4	52	42	13.6	8.0
*Prunus fasciculata*	2.0	0.5	0.2	15	15	3.1	1.5
*Salazaria mexicana*	1.9	0.5	0.3	24	24	2.2	1.1
*Senna covesii*	5.4	0.5	0.1	6	6	7.7	1.4
*Sphaeralcea ambigua*	4.9	3.0	0.6	36	24	8.3	2.5
*Stephanomeria pauciflora*	14.8	2.2	0.1	24	21	9.2	0.7
*Thamnosma montana*	6.2	1.6	0.3	24	21	6.4	1.2
*Xylorhiza tortifolia*	26.7	0.4	0.0	18	9	2.0	0.1
Versatile							
*Acamptopappus shockleyi*	0.8	0.3	0.3	18	15	1.4	2.2
*Ambrosia dumosa*	0.7	7.5	10.3	45	42	16.5	24.3
*Atriplex canescens*	0.6	0.1	0.2	12	15	1.0	1.4
*Cylindropuntia echinocarpa*	1.0	0.5	0.6	21	27	2.6	2.1
*Ephedra nevadensis*	1.0	4.2	4.1	45	52	9.3	7.9
*Ephedra viridis*	1.3	0.1	0.1	12	12	1.2	0.9
*Eriogonum fasciculatum*	1.0	0.5	0.5	24	27	1.9	1.7
*Lycium andersonii*	0.9	2.2	2.5	36	42	6.0	6.0
*Parkinsonia microphylla*	1.5	1.8	1.2	9	9	19.6	13.0
*Pleuraphis rigida*	0.7	2.0	2.9	12	21	16.6	13.6
*Yucca baccata*	0.9	0.3	0.3	12	12	2.5	2.7
*Yucca schidigera*	0.5	0.2	0.5	9	15	2.6	3.0
Decreasers							
*Ambrosia deltoidea*	0.3	1.0	3.9	9	9	11.3	42.6
*Coleogyne ramosissima*	0.1	1.0	13.9	33	39	2.9	35.3
*Cylindropuntia acanthocarpa*	0.2	0.1	0.3	18	24	0.4	1.3
*Cylindropuntia bigelovii*	0.3	0.3	1.0	9	12	3.2	8.4
*Cylindropuntia ramosissima*	0.2	0.0	0.1	9	21	0.3	0.5
*Echinocereus engelmannii*	0.1	0.0	0.1	6	15	0.1	0.6
*Ericameria cooperi*	0.5	0.6	1.4	24	27	2.7	5.1
*Grayia spinosa*	0.1	0.8	5.1	15	24	5.0	21.1
*Juniperus californica*	0.0	0.0	0.9	6	12	0.6	7.1
*Krameria erecta*	0.4	0.2	0.5	18	21	1.2	2.5
*Krascheninnikovia lanata*	0.2	0.1	0.7	18	21	0.8	3.4
*Larrea tridentata*	0.4	8.1	21.5	48	64	16.7	33.8
*Menodora spinescens*	0.3	0.2	0.6	12	15	1.7	4.0
*Prosopis glandulosa*	0.0	0.0	0.4	3	6	0.1	6.8
*Yucca brevifolia*	0.1	0.1	0.9	15	21	0.6	4.3

1 R = ratio of disturbed:undisturbed abundance; AD = mean relative abundance for disturbed areas among studies; AU = mean relative abundance for undisturbed areas among studies; OD = percent occurrence (out of 33 studies for which individual species data could be extracted) for disturbed areas; OU = percent occurrence (out of the same 33 studies) for undisturbed areas; ADP = relative abundance only when present for disturbed studies; AUP = relative abundance only when present for undisturbed studies.

**Table 5.** Relationship of time since disturbance (TSD) and annual plant community characteristics in the Mojave and Sonoran Deserts of the American Southwest.

Reference	Disturbance type	TSD (yr)	Sampling method^1^	No. yrs. sampled^1^	y^2^ = mTSD + b			Yrs. to 100%^3^
					m	b	*r*	
Cover								
Johnson *et al*. [68]	Powerline	1–6	PP	6	412.520	−149.3	0.43	1
Callison *et al*. [51]	Fire	1–37	CS	7	−8.681	420.1	−0.43	—
Brooks and Matchett [61]	Fire	6–14	CS	3	35.468	39.4	0.99	2
Vamstad and Rotenberry [67]	Fire	2–65	CS	6	−0.221	121.2	0.20	—
Richness								
Callison *et al*. [51]	Fire	1–37	CS	7	−0.897	94.3	−0.30	—
Brooks [60] - below shrub	Fire	1–4	PP	4	6.661	13.5	0.97	13
Brooks and Matchett [61] - 1 m^2^	Fire	6–14	CS	3	5.559	59.7	0.92	7
Brooks and Matchett [61] - 0.01 ha	Fire	6–14	CS	3	2.860	63.5	0.64	13
Vamstad and Rotenberry [67]	Fire	2–65	CS	6	0.132	110.1	0.41	—
Similarity								
Callison *et al*. [51]	Fire	1–37	CS	7	0.522	50.4	0.55	95
Brooks and Matchett [61]	Fire	6–14	CS	3	−5.596	92.2	−0.91	—
Vamstad and Rotenberry [67]	Fire	2–65	CS	6	0.099	52.1	0.14	480

1 CS = chronosequence, PP = permanent plot. No. yrs. represents how many different years were represented by the sampling.

2 Cover and richness are expressed as a percentage of undisturbed areas.

3 Years required for measures to reach 100% of levels on undisturbed areas, as estimated by the regression equation.

## Appendix. Summary of 47 studies meeting inclusion criteria for a quantitative analysis of plant recovery following disturbance in the Mojave and Sonoran Deserts of the American Southwest.

**Table:**

Reference^1^	Disturbance type	TSD (yr)^2^	Temporal sampling^3^	Community type^4^	Species metric^5^	Species included	
						Annuals	Perennials
Mojave Desert							
Wells [23]	Ghost town (roads, townsite)	33	–	MS	D, F		×
Rickard and Shields [42,104]	Nuclear detonation, clearing	2–3	–	MS	II	×	×
Davidson and Fox [43]	Vehicle staging area	1	–	CR	D, F	×	×
Vasek *et al*. [44]	Pipeline corridor	12	–	CR	–		×
Vasek *et al*. [45]	Powerline corridor	1, 36	CS	CR, SB	–		×
Vasek [24]	Borrow pit	9	–	CR	C, D, F		×
Lathrop and Archbold [46]	Aqueduct corridor	9, 66	CS	CR	–		×
Lathrop and Archbold [47]	Powerline, pipeline corridor	1–55	CS	CR	–		×
Webb and Wilshire [48]	Ghost town (roads, townsite)	51	–	MS	C, D		×
Lathrop [49]	Military (roads, clearing)	36	–	CR	C, D		×
Webb *et al*. [50]	Old agricultural field	20	–	CR	C, D		×
Callison *et al*. [51]	Fire	1–37	CS	BB	C	×	×
Prose and Metzger/Prose *et al*. [7,52]	Military (roads, clearing)	40	–	CR	C, D		×
Carpenter *et al*./Carpenter [31,105]	Old agricultural field	52–79	CS	CR, JT, MS	II		×
Webb *et al*. [30,20]	Pipeline corridor, ghost town	42–74	–	BB-MS	C, D		×
Medica *et al*. [53]	Fire	2–8	PP	MS	C, D	×	×
Gabbert *et al*. [54]	Clearing	6–12	–	CR, BB, MS	D		×
Minnich [55]	Fire	1–47	CS	BB, JT	C, D		×
Lei [56]	Fire	1–17	CS	BB	D		×
Walker and Powell [57]	Abandoned road	3	–	JT	D	×	×
Bolling and Walker/Bolling [27,106]	Abandoned road	5–88	CS	CR	C, D		×
Loik *et al*. [58]	Fire	1	–	JT-MS	C, F		×
Prose and Wilshire [28]	Military (roads, clearing)	22–43	–	CR	C, D	×	
Steiger and Webb [59]	Military (roads, clearing)	42	–	CR-MS	C, D		×
Brooks [60]	Fire	1–4	PP	CR	–	×	
Brooks and Matchett [61]	Fire	6–14	CS	BB	C	×	×
Webb and Thomas [62]	Ghost town (roads, townsite)	19–92	CS	MS	–		×
Webb *et al*. [63]	Fire	4–41	PP	BB-MS	C, D		×
Abella *et al*. [64]	Pipeline corridor	8, 38	CS	CR	C, D	×	×
Abella *et al*. [65]	Fire	2	–	CR-BB	C, D, F	×	×
Scoles-Sciulla and DeFalco [66]	Abandoned road	1–7	CS	CR	C		×
Vamstad and Rotenberry/Vamstad [67,107]	Fire	2–65	CS	BB-JT	C	×	×
Sonoran Desert							
Johnson *et al*. [68,108]	Powerline corridor	1–6	PP	MS	–	×	×
O'Leary and Minnich [69]	Fire	5	–	CR	F		×
Hessing and Johnson [70]	Powerline corridor	1–5	PP	SU	–		×
McLaughlin and Bowers [71]	Fire	1–2	–	SU	C, D		×
Cave and Patten [72]	Fire	1–2	–	SU	D	×	×
Brown and Minnich [73]	Fire	3–5	–	CR	C	×	×
Roundy and Jordan [74]	Plowing	12	–	SU	D	×	×
Wilson *et al*. [75]	Fire	1	–	SU	D	×	
Kade and Warren [8]	Military (roads, clearing)	56	–	CR	C, D		×
Alford *et al*. [29]	Fire	5–21	CS	SU	–		×
Abella *et al*. [76]	Fire	1–2	PP	SU	C, F	×	×
Primary succession							
Webb *et al*. [30,20]	Debris flow	5-millennia	CS	BB-MS	C, D		×
McAuliffe [77]	Debris flow	100s-millennia	CS	MS	D		×
McAuliffe [78]	Debris flow	Various^6^	CS	CR-MS	C		×
Bowers *et al*. [33]	Debris flow	5–3,100	CS	MS	C, D		×

1 Studies of secondary succession by desert are given first, then studies of primary succession (Webb *et al*. [30,20] was conducted in the Mojave Desert, McAuliffe [77,78] in the Sonoran, and Bowers *et al*. [33] in Mojave-Sonoran transitional ecosystems). Studies within these categories are arranged chronologically. Multiple references are given when more than one document reported on a study or when additional data were derived from theses associated with an article. Note that Webb *et al*. [30, 20] examined both secondary and primary succession.

2 Time since disturbance.

3 For studies that compared recovery through time, methods used were either chronosequences (CS) or permanent plots (PP).

4 BB = blackbrush (*Coleogyne ramosissima*), CR = creosote bush (*Larrea tridentata*), JT = Joshua tree (*Yucca brevifolia*), MS = mixed shrub, SB = saltbush (*Atriplex* spp.), and SU = Sonoran upland.

5 C = cover, D = density, F = frequency, II = importance index, and – = data not broken down by species, provided as a single measure of total community abundance.

6 Holocene and Pleistocene deposits were examined, with ages estimated based on the degree of soil development.
